# Minimal Residual Disease in Acute Lymphoblastic Leukemia: Technical and Clinical Advances

**DOI:** 10.3389/fonc.2019.00726

**Published:** 2019-08-07

**Authors:** Irene Della Starza, Sabina Chiaretti, Maria S. De Propris, Loredana Elia, Marzia Cavalli, Lucia A. De Novi, Roberta Soscia, Monica Messina, Antonella Vitale, Anna Guarini, Robin Foà

**Affiliations:** ^1^Hematology, Department of Translational and Precision Medicine, Sapienza University of Rome, Rome, Italy; ^2^GIMEMA Foundation, Rome, Italy; ^3^Department of Molecular Medicine, Sapienza University of Rome, Rome, Italy

**Keywords:** acute lymphoblastic leukemia (ALL), minimal residual disease (MRD), flow cytometry, real time quantitative PCR (RQ-PCR), next generation flow cytometry (NGF), digital droplet PCR (ddPCR), next generation sequencing (NGS), novel agents

## Abstract

**Introduction:** Acute lymphoblastic leukemia (ALL) is the first neoplasm where the assessment of early response to therapy by minimal residual disease (MRD) monitoring has proven to be a fundamental tool to guide therapeutic choices. The most standardized methods to study MRD in ALL are multi-parametric flow cytometry (MFC) and polymerase chain reaction (PCR) amplification-based methods. Emerging technologies hold the promise to improve MRD detection in ALL patients. Moreover, novel therapies, such as monoclonal antibodies, bispecific T-cell engagers, and chimeric antigen receptor T cells (CART) represent exciting advancements in the management of B-cell precursor (BCP)-ALL.

**Aims:** Through a review of the literature and *in house* data, we analyze the current status of MRD assessment in ALL to better understand how some of its limitations could be overcome by emerging molecular technologies. Furthermore, we highlight the future role of MRD monitoring in the context of personalized protocols, taking into account the genetic complexity in ALL.

**Results and Conclusions:** Molecular rearrangements (gene fusions and immunoglobulin and T-cell receptor-IG/TR gene rearrangements) are widely used as targets to detect residual leukemic cells in ALL patients. The advent of novel techniques, namely next generation flow cytometry (NGF), digital-droplet-PCR (ddPCR), and next generation sequencing (NGS) appear important tools to evaluate MRD in ALL, since they have the potential to overcome the limitations of standard approaches. It is likely that in the forthcoming future these techniques will be incorporated in clinical trials, at least at decisional time points. Finally, the advent of new powerful compounds is further increasing MRD negativity rates, with benefits in long-term survival and a potential reduction of therapy-related toxicities. However, the prognostic relevance in the setting of novel immunotherapies still needs to be evaluated.

## Introduction

Acute lymphoblastic leukemia (ALL) is a malignant disorder that originates from hemopoietic lymphoid precursors, that can be of B- (80–85%) or T-cell (20–25%) derivation: the acquisition of a series of genetic aberrations leads to an impaired maturation, with an arrest in the differentiation process and an abnormal proliferation (1). As a consequence, the accumulation of leukemic cells occurs in both the bone marrow, where it suppresses the physiologic hemopoiesis, as well as in extra-medullary sites.

The leukemic transformation generates a progeny of leukemic lymphoid blasts that have undergone a maturation block in an early phase of the differentiation process. The pathophysiological bases of the symptoms and signs of ALL consist in a suppression of normal hemopoiesis, in the infiltration and colonization of lymphoid organs and in the release of lymphokines and mediators of inflammation of both leukemic cells and normal cells. It is a heterogeneous malignancy also in terms of clinical manifestation and prognosis.

ALL is the most frequent cancer in childhood and is diagnosed also in adulthood, with peaks of incidence between the age of 2 and 5 years and after the age of 50 (2, 3) with 60% of cases occurring in individuals below 20 years of age (4).

Owing to the application of risk-adapted therapy and improved supportive care, the 5 years survival rate for children with ALL has significantly increased from 57 to 92% over time (5–10). However, relapses still occur in 20% of children with ALL (11) and are associated with a poor outcome (12). In adults, the frequency of high-risk leukemia and relapse risk is higher; implementing pediatric ALL treatment algorithms has led to substantial improvements in adult ALL. Nevertheless, 40–50% of adult patients still relapse (13, 14). This can be partly attributed to the higher incidence of high-risk molecular aberrations in older patients and also to the fact that older patients are less fit to tolerate intensive treatments.

ALL is the first neoplasm where the assessment of early response to therapy by minimal residual disease (MRD) monitoring has proven to be a fundamental tool for guiding therapeutic choices. At present, MRD detection is used for: the assessment of initial treatment response and subsequent definition of MRD-based risk groups with consequent risk-stratification; monitoring disease burden in the setting of stem cell transplantation (SCT); early marker of impending relapse.

## Methodologies for MRD Detection

MRD is defined as any approach—including cytogenetics, flow cytometry, PCR-based tools, and high throughput sequencing methods—aimed at detecting and possibly quantifying residual tumor cells beyond the sensitivity level of cytomorphology. To be informative, MRD assays for ALL should allow to detect one leukemic cell among 10,000 normal cells or more in virtually all patients. Currently, the most standardized methods to study MRD in ALL are: multi-parametric flow cytometry (MFC) of leukemia-associated immunophenotypes (LAIP) and, more so, polymerase chain reaction (PCR) amplification-based methods that use leukemia-specific (fusion gene transcripts) or patient-specific (immunoglobulin/T-cell receptor (IG/TR) gene rearrangements) molecular markers (15–20).

### Source of Material for MRD Evaluation

In the past, it has been debated if peripheral blood (PB), rather than bone marrow (BM), could be used for MRD monitoring, regardless of the technique used (MFC or PCR). It is nowadays clear that the scenario is different between B-lineage and T-lineage ALL: in fact, in BCP-ALL, MRD levels tend to be 1–3 logs lower in PB than in BM (21, 22) and that bone marrow assessments cannot be replaced by blood analyses; at variance, in T-ALL, it has been shown—both in children and adults—that PB is a reliable source for MRD monitoring since there are no significant differences with BM and therefore they could be used as an alternative source. Nevertheless, also in T-lineage ALL MRD assessments are normally carried out on BM samples.

### Multi-Parametric Flow Cytometry Analysis

This approach takes advantage from the presence of proteic epitopes (antigens) in the nucleus, cytoplasm or surface of the cell, which are sequentially acquired during normal cell development. Antigens are differently expressed by B- and T-lymphoblasts, and their expression is assessed by quantification of the signal emitted by fluorochrome-conjugated-specific monoclonal antibodies (MoAb). The LAIP must be identified at diagnosis, before any therapy in each ALL case, by comparing the marker profile of leukemia cells to reference bone marrow samples, through various combinations of monoclonal antibodies against surface, cytoplasmic, or nuclear leukocyte antigens. A second approach is represented by the so-called “different from normal (DFN)” analysis, which defines leukemic blasts by recognizing immunophenotypic changes with respect to a normal counterpart population (either hematopoietic progenitors of similar lineage and maturational stage) thought the evaluation of antigenic patterns expression (23). This tool has the advantage of studying MRD without the need of a diagnostic immunophenotype, but it requires standardization and needs further implementation.

During the years, antibody-conjugated fluorochromes were developed to increase the number of “colors;” further advances in the field increased the possibility of studying a great number of functionally distinct lymphocyte populations in human PB (24–26). The introduction of violet lasers (405 nm) and of nanocrystals (called quantum dots) or organic polymers capable of conducting electrons (27) led to the current MFC capable of analyzing up to 18 colors in a single cell (28). At present, the most commonly used MFC panels comprise 6–8 MoAb combinations. The refinement of MFC has required a parallel advancement in hardware, software and reagents. Engineering advances in optics and signal processing (digital electronics) are areas of active development (24, 29). Different software packages are now available, including FACSDiva^TM^ [Becton Dickinson], Kaluza^TM^ [Beckman Coulter], FlowJo^TM^ [www.treestar.com], Cytobank^TM^ [www.cytobank.org], SPICETM [http://exon.niaid.nih.gov/spice/], SamSPECTRAL^TM^ [R-package], FLAMETM, SPADE^TM^ [www.cytospade.org], FlowType^TM^ [R-package], FlowCAPTM [flowcap.- flowsite.org], GemStone^TM^ [www.vsh.com], Infinicyte^TM^ [www.infinicyt.com].

**Figure 1:**
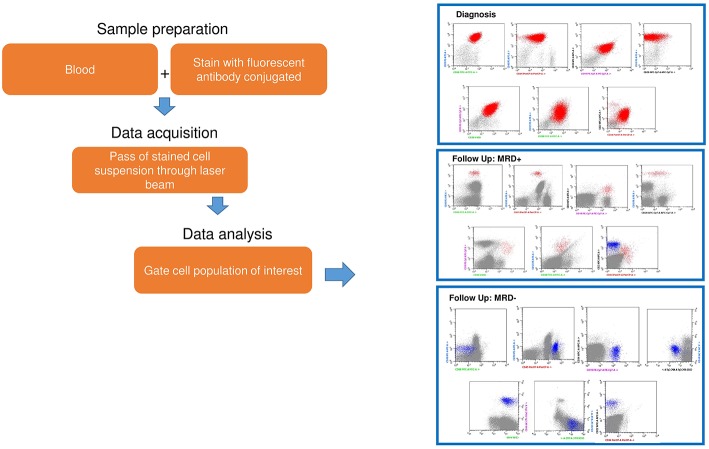
An example of standard flow cytometry MRD analysis. Sample preparation from peripheral and/or bone marrow blood requires staining with fluorescent conjugate antibodies. Data acquisition requires: (1) the stained cells through a laser beam, (2) registration of fluorescence emission from conjugate cells. This is followed by data analysis with a specific software.

Flow cytometry can be successfully applied to the majority of cases (>90%) and can reach a sensitivity of 10^−3^-10^−4^ (one leukemic cell out of 1000–10,000 normal cells) (Table 1 and Figure 1) (30). Flow cytometry analysis is quick, can release MRD evaluations in few hours, and is, therefore, also useful to assess the therapeutic response following the first 2 induction weeks (31). However, some limitations exist, such as the fact that the samples must be analyzed shortly after collection to avoid cell death, a problem when shipment is required for the centralized evaluation of MRD referral laboratories. Furthermore, (a) post-induction regeneration of normal lymphoid cells co-expressing some ALL-type antigens can lead to false positive results in B-ALL cases, (b) the bone marrow sample hypocellularity and, in some patients, phenotypic shift can induce erroneous or difficult interpretations (32, 33). The EuroFlow Consortium has optimized and standardized immunostaining protocols for the diagnosis, classification and prognostic subclassification of hematologic malignancies, as well as for the detection of MRD during the clinical follow-up; however, experienced operators are still needed to correctly evaluate MRD results (34, 35).

**Table 1:** Technical comparison of MRD standard methods.

**Method**	**Target**	**Applicability**	**Material**	**Quantification**	**Sensitivity**	**Advantages**	**Disadvantages**
Multicolor flow cytometry	Leukemia-associated immunophenotypes	>90%	Cell suspension (peripheral blood, bone marrow, needle aspirates of several tissues)	Absolute	3–4 colors:10^−3^/10^−4^ 6–8 colors: 10^−4^	Fast Widely applicable Single cell analysis Easy storage of data Information on whole population Standardized	Relatively sensitive Operator dependent Relatively expensive Cell number available
Real-time quantitative (RQ) PCR	Recurrent fusion genes	30–40%	Nucleic acid (RNA/DNA)	Related to cell line or plasmid DNA (on RNA) Related to diagnosis (on DNA)	10^−4^/10^−5^	High sensitivity Rapid Relatively easy Stable throughout treatment Well standardized on DNA Applicable to specific leukemia subgroups: BCR-ABL1 & KMT2A-AF4	Limited applicability (target-negative in >50% of patients) RNA instability Risk of contamination Limited standardization on RNA Relatively expensive on DNA
Real-time quantitative (RQ) PCR	IG/TR gene rearrangements	90–95%	Nucleic acid (DNA)	Related to diagnosis on DNA	10^−4^/10^−5^	High sensitivity Good applicability Well standardized: international guidelines for analysis and data interpretation	Dependent on ASO-primer Laborious and time consuming Affected by clonal evolution Large amount of diagnostic DNA Relatively expensive

### Molecular Analysis

#### Antigen-Receptor Gene Rearrangements

**Figure 2:**
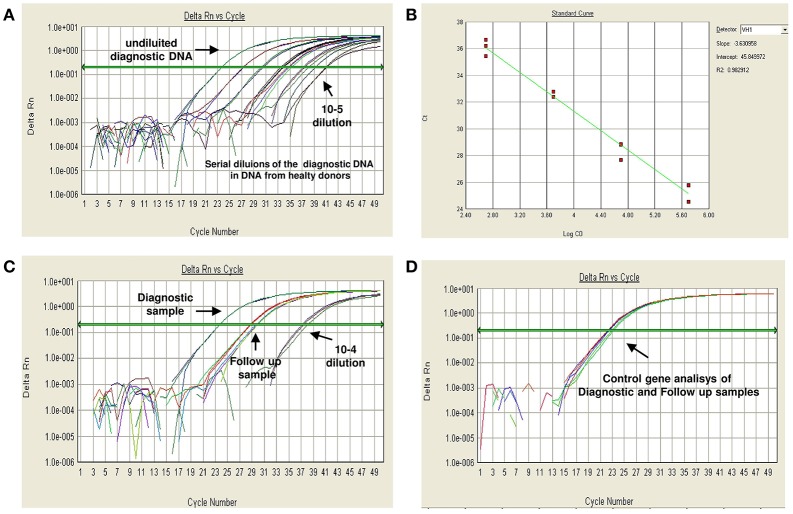
An example of RQ-PCR MRD analysis by a IGHV gene rearrangement according to EuroMRD Consortium guidelines. Patient-specific primers are used to detect malignant cells among normal lymphoid cells during follow-up. Serial dilutions of the diagnostic DNA in DNA from healthy donors are performed to verify the sensitivity and specificity of each designed primer and of each PCR assay **(A)**, and to obtain a regression curve **(B)** for the precise quantification of fluorescent levels at the single time points. Afterwards, the primer suitable for analysis is used for MRD study **(C)**. A control gene analysis (i.e., albumin) must be performed for the diagnostic and for each follow-up sample, in order to assess the amount and quality (amplificability) of the DNA in each reaction **(D)**.

The most commonly used technique is the molecular study based of antigen-receptor gene rearrangements. Immunoglobulin and T-cell receptor (IG/TR) gene rearrangements are physiological events not directly linked to the pathogenesis of the leukemia. During B- and T-lymphocytes ontogeny, the IG and TR genes are assembled by a somatic rearrangement process. The separated gene segments encoding the V, D, J regions are combined to form a single exon encoding the variable region. In this process, some nucleotides are randomly deleted or inserted at junctional sites of each segment, leading to final receptor sequences unique to each B or T lymphocytes (36). In the case of a neoplastic transformation of a single lymphoid cell, all leukemic cells will contain the same rearranged clonal IG and/or TR genes; this approach can also be exploited to detect a low number of ALL cells among a large number of normal lymphoid cells expressing gene rearrangements with different sequences. The study of these rearrangements has become the most sensitive method to assess the clonality of a lymphoid expansion. Although IG rearrangements are mostly found in B cells and TR rearrangements in T- lymphocytes, both B-lineage and T-lineage leukemic cells can display cross-lineage rearrangements, which can be used for MRD evaluation (37, 38): up to 90% of precursor B-ALL may express TR gene rearrangements (38) whereas a lower proportion of T-ALL (20%) shows IG rearrangements (39). To identify these molecular markers at diagnosis, genomic DNA derived from leukemic cells need to undergoes a PCR amplification process and the positive PCR products are then analyzed by heteroduplex or gene scan (40, 41) to establish clonality. Subsequently, clonal PCR fragments undergo Sanger sequencing to define the junctional regions and obtain complementary allele-specific oligonucleotide (ASO)-primers for MRD monitoring, mostly performed by real-time quantitative PCR (RQ-PCR). Amplification conditions and sensitivity testing for each ASO-primer are established on the diagnostic material serially diluted in normal mononuclear cells. This RQ-PCR protocol combined with fluorescently labeled probes allows the detection of up to 1 leukemic cell in 100,000 (10^−5^) normal lymphoid cells and leukemia cell dilutions are therefore used to quantify MRD in bone marrow samples collected during treatment (42) (Table 1 and Figure 2). This technology can generate at least one single sensitive molecular probe suitable for MRD analysis in over 90% of pediatric (18) and adult (43, 44) ALL patients.

Antigen-receptor gene rearrangements analysis is the most broadly applied method for MRD detection and has been extensively standardized within the EuroMRD Consortium (previously known as European Study Group- ESG-MRD-ALL) that established guidelines for the analysis and interpretation of RQ-PCR data (16) to favor an homogeneous application of MRD studies within different treatment protocols for childhood and adult ALL. Of note, a small percentage of ALL, mostly those originating from more immature cells, does not carry IG/TR gene rearrangements and occasionally technical failures can impair MRD target identification. Overall, it is not possible to perform RQ-PCR-based MRD assessments in about 5–10% of ALL cases. Another limitation of this approach is represented by clonal evolution of IG/TR rearrangement patterns during the course of the disease and at relapse, which can sometimes occur in cases with oligoclonal rearrangements leading to false negative MRD results (45). These events depend on the type of marker, disease and time to relapse (i.e., early or late relapse). The amount of diagnostic DNA is another problematic factor for this type of MRD assessment, because diagnostic DNA is needed for each MRD experiment, as well as for the detection of IG/ TR clonal rearrangements, being the quantification related to the tumor load at diagnosis. Finally, RQ-PCR is not able to define precisely the amount of residual disease in those cases in which the disease burden is very low. These cases with low MRD levels are defined as “positive-not-quantifiable” (PNQ) and their identification represents today a primary unmet need in the clinical practice when treatment decisions are based on MRD monitoring.

#### Fusion Transcripts

Another method for molecular MRD detection and monitoring is based on fusion transcript analysis. Overall, more than 40% of ALL patients carry chromosomal translocations that generate chimeric transcripts: these are potentially ideal targets for MRD assessment (46, 47), since they are main driver events, are expressed in all leukemic cells and are extremely stable during the course of the disease. Within B-lineage ALL, the most common translocation detected in adult cases is the (9, 23), also called Philadelphia chromosome, leading to the *BCR-ABL1* rearrangement (25–30% of cases); its frequency increases with age, being detected in about 50% of cases above the age of 50 years. At variance, the most common chimeric transcript in pediatric patients is represented by *ETV6-RUNX1*, that accounts for 25–30% of childhood ALL. Other fusion transcripts are *KMT2A (alias MLL)-AFF1* and *TCF3-PBX1* each accounting for 3–8% of cases, regardless of age. Infants (i.e., <1 year) carry a *KMT2A* gene rearrangement in 80% of cases. In T-ALL, *TAL1* deletions (*SIL/TAL1*) occur in about 20% of T-ALL (48). Other rarer translocations in T-ALL involve the *ABL1* gene (*NUP/ABL1, EML1/ABL1, ETV6/ABL1*). Because most of these chromosomal abnormalities have prognostic value, their detection must be performed in all cases at diagnosis (49) so that each patient can be monitored for MRD using a predefined marker throughout the course of the disease. Due to the large DNA portion in which the translocation breakpoints occurs, a patient-specific tool for MRD evaluation cannot be easily obtained; at variance, the RNA splicing process produces in all patients the same fusion transcript or few splicing variants. Thus, RNA is the optimal starting material to detect these lesions, allowing the use of a small number of quantitative-reverse transcriptase PCR (QRT-PCR) assays (50). This offers the opportunity to apply the same primer set to all patients bearing the same translocation, leading to an easy and rapid fusion transcript evaluation at diagnosis and during treatment (47).

Quantification of the fusion gene using RNA samples is achieved by comparing the amplified product to a standard curve derived from the amplification of serial dilutions of a cell line or plasmid DNA (i.e., *BCR-ABL1*+). This highly sensitive MRD assay is capable of detecting up to 1 leukemic cell within 100,000 (10^−5^) normal lymphoid cells, is not patient-specific, is relatively easy to perform and is not expensive (Table 1). However, the accuracy of this assay is hampered by the variability in the number of RNA transcripts per leukemic cell from patient to patient, and among different cells within the same leukemic clone. A full standardization is still not available and the EuroMRD Consortium is setting up guidelines for the correct interpretation of quantitative data (51).

At variance from the *BCR/ABL1* rearrangement, in patients displaying other recurrent chromosomal translocations (i.e., *KMT2A* gene rearrangements and *SIL/TAL1*), quantification of the fusion gene is well standardized within the EuroMRD Consortium (52, 53).

### New Methods for MRD Detection and Monitoring: Next-Generation Flow Cytometry, Digital-Droplet-PCR, Next Generation Sequencing

The advent of RQ-PCR (54) has represented a significant advancement with respect to conventional PCR based methods. However, the measurement of a dynamic process, such as the rate of target amplification, carries some intrinsic fluctuations that cannot be to fully eliminated: (a) non-specific amplification of spurious IG/TR rearrangements are hardly distinguishable from cases positive at a very low level (PNQ) in RQ-PCR, with an intrinsic risk of false positive/negative MRD detections; (b) the use of RQ-PCR can be limited by the lack of sufficient diagnostic material since the method is based on the comparison, for each experiment, with a standard curve based on neoplastic DNA collected at the onset of the disease, and this can limit the possibility of monitoring patients over time.

The novel **next generation flow (NGF)-MRD approach** takes advantage of innovative tools and procedures recently developed by the EuroFlow Consortium for sample preparation, antibody combinations (including choice of type of antibody and fluorochrome), and identification of B-cell precursor (BCP) pathway in the BM, which allows to define the degree of immunophenotypic deviation of BCP-ALL cells from normal BCP (also in regenerating BM). Also for T-ALL a comparable strategy is used to obtain reliable (evidence-based) antibody combinations, in order to discriminate from various types of normal T cells and other cells with cross-lineage marker expression (55, 56).

NGF-MRD is faster and reproducible, it has a greater applicability (>95%). Moreover, the costs of reagents and assays are estimated to be lower than those of NGS (57). However, it requires fresh material analyzed within 24 h after sampling. Finally, NGF-MRD strategies provide a full insight into the composition of normal cells and aberrant cells, and can help to better characterize ALL cell population changes such as treatment-induced immunophenotypic shifts (58, 59), heterogeneity in the blast cell population with a de-differentiation to immature stem like-cells and aberrations in other lineages.

**Figure 3:**
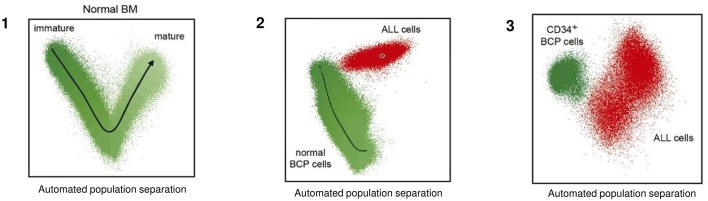
Schematic representation of normal and malignant B-cell precursor by multidimensional analysis based on EuroFiow-based NGF-MRD. This analysis is not based on a single marker but on multiple required antigens, allowing to define the degree of immunophenotypic deviation of BCP-ALL cells from normal BCP, visualized in multivariate analysis plots. **(1)** Representation of Automated population separation (APS) of physiological phases of B-cell maturation. **(2)** Plot of ALL cells (red dots) with respect to normal BCP cells (green dots). **(3)** Plot of ALL cells (red dots) with respect to immature CD34+ BCP cells only (green dots). Adapted from van Dongen et al. (60).

A similar applicability associated with a significantly increased sensitivity for the novel EuroFlow-NGF approach vs. conventional flow-MRD has been described in multiple myeloma (59); NGF-MRD reaches a sensitivity close to 10^−6^, while conventional flow tools reach sensitivities in the range of 10^−4^-10^−5^ (Table 2 and Figure 3). The greater sensitivity of NGF-MRD is mostly due to the use of standardized approaches, including instrument setting, sample processing with bulk lysis procedure, immunostaining, data acquisition, and data analysis with standardized (even automated) gating strategies for definition of cell populations (59). However, the acquisition of a large number of cells is needed to reach the required sensitivity.

**Table 2:** Technical comparison of MRD novel methods.

**Method**	**Target**	**Applicability**	**Material**	**Quantification**	**Sensitivity**	**Advantages**	**Disadvantages**
NGF	Leukemia-Associated Immunophenotypes	>95%	Cell suspension (peripheral blood, bone marrow, needle aspirates of several tissues)	Absolute	10^−4^/10^−6^	High sensitivity High applicability Fast and reproducible Accurate quantification Highly standardized with possibilities for automated gating	Education and training required Many cells needed to reach the required sensitivity Requires fresh material analyzed within 24 h after sampling Expensive
ddPCR	IG/TR gene rearrangement	90–95%	Nucleic acid (DNA)	Absolute	10^−4^/10^−5^	High sensitivity Good applicability (90-95%) no need of standard curve easy	Dependent on ASO-primer No standardized: no guidelines for analysis and data interpretation Available in few labs Relatively expensive
NGS	IG/TR gene rearrangements	>95%	Nucleic acid (DNA)	Absolute	10^−4^/10^−6^ (depending on amounts of DNA analyzed)	High sensitivity High applicability (>95%) Potential to identify clonal evolution Provides information on background repertoire of B and T cells Not dependent on ASO-prime	No standardized: no guidelines for analysis and data interpretation Available in few labs Discrimination from normal clonal background Need of a bioinformatic analysis Expensive

In the forthcoming decade, the new flow technologies will improve applicability and specificity of flow MRD measurements.

**Figure 4:**
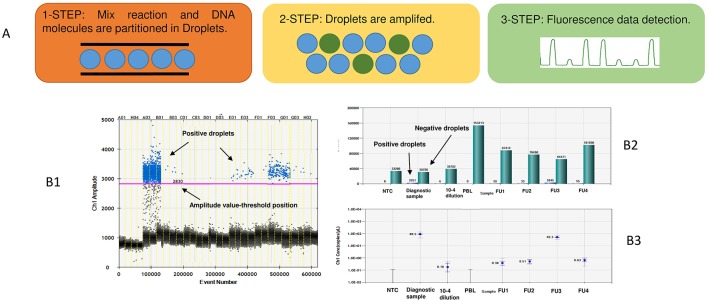
An example of ddPCR MRD analysis. In the **(A)** is reported a schematic diagram of a ddPCR experiment. 1-Step: the mix reaction is prepared with the same primer/probes of the TaqMan assay. Both the reaction and the DNA samples are partitioned into 20,000 droplets of identical volume through a microfluidic system. 2-Step: in a thermal cycler 20,000 PCR reactions are amplified and fluorescence is the output during the reaction of polymerization. 3-Step: a droplet reader analyzes each droplet individually and detects an increased fluorescence in positive droplets, which contains at least one copy of target DNA. **(B)** Each droplet is plotted on the graph of fluorescence intensity vs. droplet number **(B1)**. The concentration is calculated on the fraction of empty droplets (green bar) that are the fraction that does not contain any target DNA by software **(B2)**. Fraction of positive droplets is fitted to a Poisson algorithm to determine absolute copy number, results are presented in copies per 1-μL **(B3)**.

The **digital PCR technology (ddPCR)** (61) based on sample partitioning (mimicking limiting dilution) and Poisson statistics, has the potential to overcome the limitations of RQ-PCR. DdPCR (62, 63) is the third-generation implementation of conventional PCR that allows the quantitation of nucleic acid targets without the need of the calibration curves (64). As reported in several studies (65, 66), based on the dynamic nature of two methods, ddPCR appears more accurate than RQ-PCR since: (i) each sample is partitioned in droplets and each droplet is analyzed individually, (ii) small changes in fluorescence intensity are more readily detected, and (iii) the ratio between target DNA molecules to PCR reagents is substantially higher; this increases its amplification efficiency (67). Finally, the presence of inhibitors negatively affects the RQ-PCR efficiency but not that of ddPCR (68) (Table 2 and Figure 4).

Moreover, RQ-PCR, as an exponential process, is able to greatly amplify even small differences in reaction efficiency, leading to discrepancies in the final results with 68% confidence intervals (http://www.biorad.com/webroot/web/pdf/lsr/literature/Bulletin_6407.pdf).

**Figure 5:**
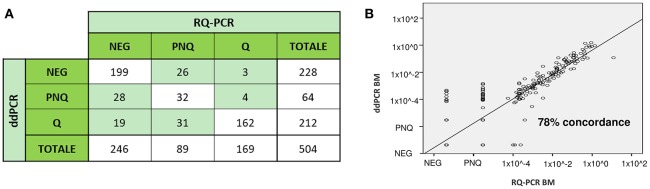
MRD analysis by both RQ-PCR and ddPCR in 504 follow up samples from 176 patients with several hematological malignancies (Acute Lymphoblastic Leukemia, *n* = 80, Follicular Lymphoma, *n* = 48, Chronic Lymphocytic Leukemia, *n* = 40, Mantle Cell Lymphoma, *n* = 8). The study was performed on bone marrow (BM) and peripheral blood (PB) samples, based on the material availability. MRD detection was concordantly positive or negative in 78% (393/504) of FU samples (r = 0.78, *P* < 0.0001), while 22% (111/504) were identified as discordant **(A)**. Most of the discordances occurred in FU samples with a low level of disease - positive not quantifiable or negative—and did not appear to cluster in specific disease subsets. Overall, the use of ddPCR significantly reduced the proportion of PNQ samples compared to RQ-PCR (64/504 [13%] vs. 89/504 [18%], respectively) (*p* = 0.03), increasing the proportion of Q samples (212/504 [42%] vs. 169/504 [33.5%], *p* = 0.006). In **(B)** is reported the concordance rate (78%) between the two methods on all BM samples analyzed (unpublished data). Q, positive and quantifiable; PNQ, positive and not quantifiable; NEG, negative.

ddPCR is an endpoint measurement with 95% confidence intervals, as reported by the manufacturer's application guide. The ddPCR technology has been applied to various fields of medical diagnostics, in particular in molecular oncology (62, 64, 65) and in prenatal diagnosis (69, 70). Several reports on the use of ddPCR in hematologic malignancies (71–74) are available in the literature. Three recent articles, including two from our Center, have compared ddPCR to RQ-PCR in adult patients affected by mature lymphoid malignancies and in Ph- ALL (75–77). These studies have established analytical parameters to investigate the applicability of ddPCR for MRD detection and concluded that ddPCR has a sensitivity, accuracy and reproducibility at least comparable to that of RQ-PCR. Regarding MRD evaluations, some discordances have been observed at very low disease levels; in this setting, ddPCR showed a good analytical performance to quantify those low positive samples defined as PNQ by RQ-PCR or to identify the false MRD+ cases. These results have been confirmed from our group in a wider comparative analysis including 175 patients with different lymphoid malignancies (78) (Figure 5). Recently, the clinical significance of ddPCR has been reported in a pediatric cohort of ALL patients (79). The authors showed that among “slow early responder” patients, most relapses occurred in cases with quantifiable ddPCR MRD at day +78, while patients with a negative or PNQ MRD by ddPCR at day +78 had a better outcome, emphasizing that high-risk treatment could be offered only to ddPCR quantifiable cases.

No established guidelines for ddPCR MRD analysis and interpretation have so far been defined. A major standardization effort is underway within the EuroMRD Consortium.

Several groups have shown the value of **next-generation sequencing (NGS**) technologies for MRD detection in precursor and mature B-cell tumors (80–82) NGS can be used to detect clone-specific IG/TR index sequences; clonal sequences detected at diagnosis can be re-detected and quantified in each follow-up sample. By using universal primers, this method allows to monitor all IG/TR gene rearrangements at the same time, providing a complete picture not only of the residual leukemia but also of the normal immune repertoire (82).

**Figure 6:**
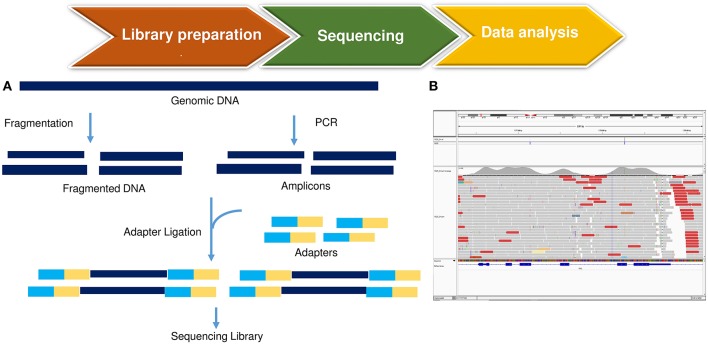
An example of NGS MRD analysis. **(A)** starting from genomic DNA, a library is prepared by fragmentation and conjugation with adaptive sequences, composed with few nucleotides. The library is subsequently amplified and sequenced, with the production of so-called ≪reads≫. **(B)** Data analysis is performed through the use of bioinformatic tools, that align experiment-derived reads to a reference genome.

Sensitivity is a critical aspect in MRD detection. Methods allowing a sensitivity higher than 10^−5^ (routinely achieved by RQ-PCR) might be of interest to identify very low-level disease. Studies using the NGS platform in ALL and chronic lymphocytic leukemia have demonstrated that a sensitivity level of 10^−6^ (81, 83) is achievable when higher amounts of DNA are used (Table 2 and Figure 6). This is reflected in the possibility of detecting early clonal evolution, a relatively frequent occurrence in relapsed ALL (84).

Many authors have reported that NGS appears more specific than RQ-PCR in predicting relapse in ALL patients after induction (82) as well as after allogenic SCT (85). A comparative MRD analysis between RQ-PCR and NGS showed within the Berlin Frankfurt Münster (BFM) trials a change in the stratification risk, mainly due to different interpretations of the two techniques within low-positive samples (82). The NGS quantitative discrimination is always superimposable to the sensitivity, whilst the RQ-PCR quantitative range is usually inferior to the sensitivity threshold leading to cases which are defined as PNQ, as previously described (81). Another comparative analysis, performed at our Center, between RQ-PCR, ddPCR and NGS has documented a more precise prediction of relapse with the new methods compared to the standard technique and a change in the adult ALL risk stratification (86). However, NGS has a substantial intrinsic complexity and involves major costs. Furthermore, at the moment robust and broadly applicable NGS-based MRD standardized protocols are still not available in academic laboratories. The Euroclonality-NGS Consortium is working to standardize guidelines for analysis and data interpretation.

Through the integration of these technologic innovations, we are moving toward a system that can quickly integrate all the information necessary for a more precise evaluation of the response to treatment in ALL patients. ddPCR and NGS appear to be feasible and attractive alternative methods for MRD assessment that can help to classify more precisely cases that RQ-PCR is unable to detect or quantify.

## Clinical Significance of MRD

### MRD in All Without Major Molecular Transcripts

The clinical impact of MRD is now widely accepted and is regarded today as the most important prognostic factor in the state-of-the-art management of ALL. MRD can provide different information, according to the timing in which it is performed (very early during treatment, after induction/consolidation, before and after SCT) and, more recently, it can be refined by the evaluation of additional genomic markers.

It is now widely acknowledged that MRD detection should be carried out with molecular methods, as it occurs in European countries; at variance, in the United States MFC, with few exceptions, is more commonly used, also for the lack of standardized guidelines for molecular analysis and data interpretation.

In the modern era, treatments include combination chemotherapy for the achievement of a complete hematologic and clinical remission (CR), followed by post-remission consolidation therapy with or without SCT, plus an effective central nervous system prophylaxis. Several pediatric and adult ALL study groups have established informative checkpoints in their respective treatment protocols. Among these, the initial MRD response to therapy is a relevant prognostic factor in both childhood and adult ALL (87, 88); indeed, MRD negativity at very early time points during induction therapy correlates with a particularly good outcome both in childhood and adult ALL, and is indicative of a high sensitivity to chemotherapy. In children, it has been shown that MRD negativity achieved as early as at day 15 correlates with an excellent outcome; in adults, the early evaluation of MRD is usually carried out at a later timing (i.e., end of induction, week 4) and it again correlates with better survival rates. Some studies have reported that patients with a very rapid tumor clearance after 2 weeks of therapy have a very good prognosis (31, 89). Along the same line, in a MFC-based trial, high levels of disease at day 14 of treatment (>30%) identified a small subgroup of patients with a particularly poor prognosis and a median event-free survival (EFS) and overall survival (OS) of only 9 and 21 months, respectively; however, MRD evaluation was not significant if evaluated at CR achievement (90). It must be again underlined that in European protocols the use of MFC for MRD evaluation is normally substituted by molecular techniques.

The other time points that hold prognostic significance are the end of induction/early consolidation. Indeed, the first pivotal study on molecular MRD analysis was carried out by the German Multicenter Study Group for Adult ALL (GMALL) on a large cohort of Ph- patients with standard-risk and high-risk features (*n* = 580 in CR) that showed that the molecular response to standard induction and consolidation treatment was the only significant prognostic factor for remission duration and survival in both risk groups (91). These data have been confirmed by others groups, regardless of the cut-off values, MRD technique, timing of MRD analysis and the target patient population chosen (92–94). The PETHEMA group evaluated the role of MRD (by flow-cytometry, cut-off: 5 × 10^−4^) in 326 adult high-risk Ph- ALL and confirmed that the only prognostic factor was represented by MRD persistence after induction and early consolidation (92). The GRAAL conducted an analysis on 955 patients to assess the role of SCT, taking into account also MRD after induction (week 6, cut-off: 10^−3^), and again showed that the role of MRD persistence is not abrogated by transplant procedures and that MRD-negative patients could be spared this approach (93). The Northern Italian Study Group (NILG) performed MRD evaluations starting from the end of induction (week 4) and then at weeks 10, 16, and 22 in an adult patient population to assess the feasibility and efficacy of liposome-encapsulated cytarabine for central nervous system prophylaxis along with the use of lineage-targeted systemic methotrexate blocks (2.5 and 5 g/m^2^ in B- and T-lineage ALL, respectively) and other intensive pediatric-like elements (94). The early MRD response at weeks 4 (end of induction) and 10 had a profound prognostic effect. The relapse risk was very low (17% at 5 years) in the group of week 4 MRD responders and significantly lower (28%) than in non-responders (57%) when week 10 MRD results were examined. Similar results have been preliminary confirmed in the subsequent GIMEMA LAL 1913 (95).

Pivotal studies on the clinical role of MRD in Ph-ALL are summarized in Table 3.

**Table 3:** Pivotal studies on MRD prognostic value on Ph– ALL.

**Study**	**MRD methodology and cut-off**	**MRD time points**
Basso et al. (31)	Flow cytometry	Day 15
UKALL XII/ ECOG2993 trialPatel et al. (96)	RQ-PCR of Ig/T-cell receptor gene rearrangements, among others; MRD–: RQ-PCR <10^−4^	After phase 1 and 2 induction and after intensification
Joint analysis of EWALLGiebel et al. (97)	Flow cytometry or PCR-based; MRD–: <0.1% of bone marrow cells	Before HSCT
GMALLGökbuget et al. (91)	RQ-PCR of Ig/T-cell receptor gene rearrangements; MRD– with assay sensitivity of ≥10^−4^	Day 71 and at week 16
NILG-ALL 09/00 trialBassan et al. (98)	RQ-PCR of Ig/T-cell receptor gene rearrangements; MRD–: <10^−4^	Weeks 16 and 22
PETHEMA ALL-AR-03 trialRibera et al. (92)	Flow cytometry; MRD-: 5 × 10^−4^	After induction and early consolidation
Salah-Eldin et al. (99)	RQ-PCR of clonally rearranged Ig; MRD– with assay sensitivity of ≥10^−3^	After induction and after consolidation
GRAALL trialsDhèdin et al. (93)	RQ-PCR of Ig/T-cell receptor gene rearrangements; MRD–: ≤ 10^−3^	Week 6
NILG 10/07 trialBassan et al. (94)	RQ-PCR of Ig/T-cell receptor gene rearrangements; MRD–: <10^−4^	Week 10
Salek et al. (89)	Flow cytometry or PCR-based; MRD–: <10^−4^ with assay sensitivity of ≥10^−4^	At the beginning of each chemotherapy cycle and at the end of the second induction cycle
Short et al. (90)	Flow cytometry; MRD– with assay sensitivity of 0.01%	At the time of CR or CRp and again 3 months later
Berry et al. (100)^*^	Flow cytometry or PCR-based; MRD–: ≤ 0.01%	After induction and after consolidation
Gökbuget et al. (88)	RQ-PCR of clonally rearranged Ig or Flow cytometry; MRD–: <10^−4^ by RQ-PCR or ≤ 10^−3^ by flow cytometry	*Baseline MRD status*, defined as MRD persistence or MRD reappearance

* *Based on metanalysis of previously published data*.

### MRD in Ph+ All

The Ph chromosome, leading to the *BCR/ABL1* rearrangement, defines the more frequent high risk ALL subset in adults. It is found in 25–30% of patients and its incidence increases with age progression (101). Ph+ ALL was previously regarded as the subgroup with the worse outcome: the introduction of TK inhibitors (TKI) such as imatinib, dasatinib, and ponatinib (102–106), has led to the achievement of a CR in virtually all patients, to improve disease-free survival (DFS) and OS, and to increase the percentage of patients who can undergo a SCT. A common therapeutic approach for adult Ph+ ALL patients is based on the use of a TKI, with or without systemic chemotherapy for CR induction, followed by consolidation and when possible SCT. As in Ph- ALL, MRD has an important role in the management of this disease. The GIMEMA has clearly shown that the degree of MRD reduction correlates with improved DFS, regardless of the inhibitor used (107). Lee and colleagues showed that also the timing of MRD clearance is important for patients' stratification: in fact, patients who display a very early clearance have a significantly better outcome (108).

Pivotal studies on the clinical role of MRD in Ph+ ALL are summarized in Table 4.

**Table 4:** Pivotal studies on MRD prognostic value on Ph+ ALL.

**Study**	**MRD methodology and definitions**	**MRD time points**
Foà et al. (103)	RQ-PCR for BCR-ABL1 transcript and flow cytometry; Molecular response defined as a BCR-ABL1/ABL1 <10^−3^; Flow cytometry sensitivity: 0.01%;	Days 22, 43, 57, and 85
Mizuta et al. (109)	RQ-PCR for BCR-ABL1 transcript (sensitivity at least of 10^−5^); MRD–: BCR-ABL1/GAPDH <10^−5^	Before and at HSCT
Lee et al. (108)	RQ-PCR for BCR-ABL1 transcript; MRD– [BCRABL1/ABL1 ratio ≤ 0.1%] or CR [undetectable BCR-ABL1]	After 2 courses of chemotherapy, before HSCT
Pfeifer et al. (106)	RQ-PCR for BCR-ABL1 transcript; Low MRD level defined as a BCR–ABL1/ABL1 ≤ 10^−4^	After HSCT
Ravandi et al. (110)	RQ-PCR for BCR-ABL1 transcript, flow cytometry and IGH-PCR: Major molecular response (MMR) defined as a BCR-ABL1/ABL1 <0.1%; IGH-PCR sensitivity: ~0.2–1%. Flow cytometry sensitivity: 0.01%;	At the end of induction and at ~3 months intervals thereafter
GIMEMA 1509 trialChiaretti et al. (111)	RQ-PCR for BCR-ABL1 transcript; Complete molecular response (CMR) defined as BCR-ABL1/ABL1 = 0	Day 85
Kim et al. (112)	RQ-PCR for BCR-ABL1 transcript (sensitivity at least of 10^−5^); MRD–: BCR-ABL1/GAPDH ratio < = 10^−5^	Every 3 months from CRh until end of maintenance therapy
Chalandon et al. (102)	RQ-PCR for BCR-ABL1 transcript; Molecular CR defined by the absence of detectable MRD with a sensitivity of at least 0.01%	After cycle 1 and cycle 2
NILG 09/00 and 10/07 trialLussana et al. (113)	RQ-PCR for BCR-ABL1 transcript; MRD–: BCR-ABL1/ABL1 <1 × 10^−5^	Before HSCT
Ravandi et al. (114)	RQ-PCR for BCR-ABL1 transcript, flow cytometry and IGH-PCR: Major molecular response (MMR): BCR-ABL1/ABL1 <0.1%; IGH-PCR sensitivity: ~0.2–1%. Flow cytometry sensitivity: of 0.01%.	Day 21 and then every 2–3 cycles during the intensive phase, and approximately every 3 months during the maintenance phase
Nishiwaki et al. (115)	RQ-PCR for BCR-ABL1 transcript (sensitivity at least of 10^−5^); MRD–: BCR-ABL1/GAPDH ratio <10^−5^	Within 30 days prior to HSCT
Chiaretti et al. (107)	RQ-PCR for BCR-ABL1 transcript; Complete molecular response (CMR) defined as BCR-ABL1/ABL1 = 0	Day +35, +50, and post consolidation
Yoon et al. (104)	RQ-PCR for BCR-ABL1 transcript; MMR was defined as BCR-ABL1/ABL1 ≤ 0.1% for p210 or a reduction in the BCR-ABL1 transcript level by at least 3-log for p190. Complete molecular response defined as the absence of detectable BCR-ABL1transcripts	During TKI-based chemotherapy, before HSCT

In Ph+ ALL, MRD can also drive therapeutic decisions: indeed, a persistent MRD positivity and/or its reappearance can underlie the presence of resistant mutations, particularly the T315I for which alternative approaches, including novel TKIs (namely ponatinib) and/or therapies based on the combination of TKI with immunotherapeutic strategies, particularly blinatumomab have been evaluated (116).

An open issue in this setting is represented by the cases who are persistently MRD negative; in fact, while in the past SCT was considered mandatory in all Ph+ ALL patients, if clinically fit, it is currently debated if these patients might be spared this procedure, in line with the clinical policy routinely applied to Ph- ALL (102–107, 113, 115).

Finally, there is growing interest in evaluating MRD using more than one marker: in pediatric Ph+ ALL, Hovorkova and colleagues (53) showed that roughly 20% of children have significantly higher BCR/ABL1 levels (assessed by evaluating both genomic DNA and RNA fusion levels) than IG/TR or *IKZF1* deletions, indicating that the BCR/ABL1 signal arises from other hemopoietic cells. Along the same line, Cazzaniga et al. (117) showed that MRD positivity after induction and consolidation (evaluated by IG/TR) is strongly associated with relapse. A formal comparison between IG/TR and BCR/ABL1 evaluation showed comparable results in terms of relapse risk: however, concordance between the two techniques was only 69% and, again, BCR/ABL1 levels were significantly higher than IG/TR. In the adult setting, Clappier et al. (118) evaluated IG/TR and BCR/ABL1 levels (using genomic DNA and RNA) and again proved that there is an overall poor concordance between genomic DNA *BCR/ABL1* and IG/TR results (correlation coefficient = 0.51), while a good concordance is detected between genomic and RNA BCR/ABL1 levels (correlation coefficient = 0.8). Furthermore, the authors showed that there are two subsets of patients: the first group with concordant MRD results and the second displaying discordant results among IG/TR and BCR/ABL1, with BCR/ABL1 levels being higher. Interestingly, discordant cases harbor more often the p210 protein isoform, and have less frequently *IKZF1* deletions, again suggesting that the BCR/ABL1 signal derives from other cells rather than lymphoblasts and that these cases might resemble a “CML-like” subset. At present, however, it is not defined which marker is most suitable for therapeutic decisions.

### MRD and Stem Cell Transplantation

As mentioned, MRD after induction/early consolidation is the most important decision-making parameter for on allogeneic transplant, a procedure still aggravated by transplant-related mortality and toxicity, observed in about 20% of patients (119, 120). In addition, several studies have analyzed the prognostic impact on outcome of a MRD (+) status at the time of SCT (121, 122) and others have shown the prognostic relevance of pre-transplant MRD assessment in adults (98, 101, 123). In particular, Bassan et al. (98) showed that patients with MRD levels ≥10^−3^ at week 16 and/or week 22 (i.e., after consolidation) had a worse post-transplant outcome with a 6 years relapse incidence of 64% compared to 23% in patients with MRD levels <10^−3^. A recent metanalysis on 21 published reports, including over 2,000 patients, confirms that a pre-transplant positive MRD is a significant negative predictor of relapse-free survival (RFS), event-free survival (EFS), and OS; as expected, a positive MRD prior to transplant was not associated with a higher rate of non-relapse mortality (124). Taken together, these results show that MRD evaluation before transplant is extremely useful for treatment intensification, since we can offer the opportunity to adequately use immunotherapeutic compounds (e.g., blinatumomab, inotuzumab, and in the future possibly CAR-T cells) aimed at obtaining a MRD negative status.

With regard to the post-transplant setting, MRD monitoring has been much less frequently used after SCT because donor chimerism monitoring provides an alternative for early relapse detection; nevertheless, it has been shown that a IG/TR-based MRD assessment allowed an earlier and more specific detection of an impending relapse compared to chimerism analysis, showing that MRD positivity was an independent significant predictor of risk of relapse (125). Another MFC-based trial showed that patients with evidence of MRD after SCT had significantly worse outcomes compared to patients without evidence of MRD (126). In the pediatric context, pre-SCT MRD resulted the only independent prognostic factor in a multivariate analysis and after-SCT MRD is considered a reliable marker for early detection of impending relapses (127, 128).

### Relapse and Clonal Evolution

At relapse, molecular evaluation of IG/TR markers previously used for MRD monitoring might be useful to confirm the persistence of the same clone. However, it has some potential pitfalls, mostly represented by the phenomenon of clonal evolution. The analysis of all molecular markers at first diagnosis and at the time of relapse may reveal a different origin of the predominant clone (84, 129). Indeed, the loss of a molecular marker at relapse, or the expansion of a clonal marker expressed at the subclonal level at diagnosis, is a relatively frequent event: therefore, MRD assessment should be carried out with several targets (84). Finally, it must be reminded the clonal evolution can vary according to the site of relapse (medullary or extramedullary).

### MRD and Novel Markers

The extensive characterization of the genetic bases of ALL is leading to an attempt of combining MRD with other markers: a pivotal study was carried out by the GRAAL group (130) which evaluated 423 young adults with Ph- ALL in first remission (both B- and T-lineage ALL), demonstrating that a higher risk of relapse was associated with MRD persistence and can be further refined by the presence of *IKZF1* deletions in B-lineage ALL, and by the absence of *NOTCH1/FBXW7* mutation, and/or by the presence of *N/K-RAS* mutation and/or *PTEN* gene alteration in T-cell ALL.

Likewise, in childhood ALL, the generation of a risk score based on the combination of MRD at day 33, the presence of *IKZF1* intragenic deletion and *P2RY8-CRLF2*, which proved more discriminative of outcome that MRD evaluation alone, has been recently reported (131).

### MRD and Novel Agents

Novel therapies, such as monoclonal antibodies, bispecific T-cell engagers, or chimeric antigen receptor T cells (CART), are an exciting advancement in the immunotherapeutic treatment of relapse/refractory BCP-ALL. These new therapeutic approaches make MRD an almost perfect therapeutic target, considering that MRD+ patients harbor significantly less leukemic cells and therefore a more manageable clinical profile than cases in hematologic relapse.

#### Blinatumomab

Blinatumomab is a bispecific anti-CD19 and anti-CD3 construct recruiting cytotoxic T cells against CD19+ blast T cells. Blinatumomab can bridge malignant B cells directly to CD3-positive T cells, bypassing TCR specificity and major histocompatibility complex (MHC) class I molecules (132, 133) and induces T-cell activation, release of inflammatory cytokine production, specifically IL-2, IFN-γ, TNF-α, IL-4, IL-6, and IL-10 (134). Blinatumomab is the first antibody approved for treatment of refractory ALL and, more recently, for the treatment of MRD+ patients. In relapsed/refractory ALL, blinatumomab has led to morphologic responses ranging from 43 to 69% of patients (135, 136) with 76 to 88% of responding patients being MRD negative. Patients who achieved a negative molecular MRD status had a longer survival than patients who remained MRD positive (137, 138). In MRD+ patients, blinatumomab induced a complete MRD response in 78% of cases and, as expected, MRD responders has a longer RFS than non-responders. A small fraction of complete MRD responders did not undergo transplant and is still in continuous CR (139, 140).

#### Inotuzumab Ozogamicin

Inotuzumab ozogamicin is a conjugated antibody-drug (ADC) consisting of a monoclonal antibody (mAb) directed to CD22, an antigen present on the cancer cells of almost all patients with B-cell precursor ALL, linked to a cytotoxic agent. When inotuzumab ozogamicin binds to the CD22 antigen on B cells, it is internalized in the cell, where the cytotoxic agent, calicheamicin, is released to destroy the malignant cell. The drug is approved for the treatment, in monotherapy, of relapsed/refractory adult ALL patients CD22-positive. Patients treated with inotuzumab ozogamicin reached response rates ranging from 58 to 81%, with 72–78% of these having MRD results below 0.01% (141, 142) by flow cytometry assessment. While this compound appears to be extremely effective in reinducing responses, it must be underlined that CR duration is usually short, and therefore SCT must be performed as soon as possible.

#### CAR-T

CAR-T cells are patient or, less frequently, donor-derived normal T cells molecularly engineered to express a T-cell receptor mediating cytotoxicity toward anti-CD19 (in most cases). After CAR-T cells are infused into a patient, they act as a “living drug” against cancer cells: they bind to the target, become activated, proliferate and exert their cytotoxic activity. Several groups have shown that most of the responding patients (both children and adults) become MRD negative (at least by FCM) (143–145) and maintain this status for several months or years (146, 147). Data on the prognostic value of MRD in this setting are still preliminary. However, differently from first-line chemotherapeutic approaches, relapse is observed also in patients reaching a MRD negativity, mostly because of the loss of CD19. Therefore, MRD response in this setting seems to be an essential but not sufficient criterion for the definition of long-term remissions. Higher sensitivities or earlier MRD assessments might be necessary to identify a subgroup of patients with a particularly rapid and deep MRD response and a better prognosis.

## Conclusions

MRD is a powerful and independent predictor of outcome in both children and adult ALL, during treatment, in the pre- and post-SCT settings, with different prognostic meanings on the base of the clinical context.

Molecular rearrangements (gene fusion and IG/TR gene rearrangements) are widely used as targets to detect residual leukemic cells in ALL patients, although new molecular markers could be used for prognostic and therapeutic purposes, also to improve the number of evaluable patients. The alterations of IKZF1 and FLT3-ITD, might potentially represent new MRD molecular targets

Technically, MFC and RQ-PCR are the most broadly applied consolidated methods for MRD monitoring, although they present intrinsic limitations that must necessarily be overcome. ddPCR, NGS, and NGF, the standardization of which is underway, have shown to be important tools to evaluate MRD in the research setting, and will probably be soon incorporated in clinical trials due to their ability to overcome the limitation of standard approaches.

At present, the advent of powerful new agents such as ponatinib for Ph+ ALL and novel immunotherapeutic agents for B-lineage ALL (blinatumomab, inotuzumab ozogamicin) and CAR-T cells are further improving CR and MRD negativity rates, with benefits in long-term survival and a potential reduction of therapy-related toxicities. However, the prognostic relevance of MRD in the setting of novel immunotherapies still needs to be evaluated.

In the next future, MRD will certainly be the main tool to design innovative treatment algorithms including immunotherapeutic strategies and possibly sparing chemotherapy/transplant with the final aim of curing always more patients with ALL.

## Author Contributions

ID wrote the manuscript. SC wrote the manuscript and critically revised the manuscript. MD performs flow cytometry analysis. LE performs molecular analysis of fusion genes by RQ-PCR and ddPCR. MC, LD, and RS perform molecular analysis of antigen-receptor gene rearrangements by RQ-PCR and ddPCR. MM performs next generation sequencing analysis. AV provides clinical data. AG and RF critically revised the manuscript.

### Conflict of Interest Statement

The authors declare that the research was conducted in the absence of any commercial or financial relationships that could be construed as a potential conflict of interest.
